# Atypical Presentation of IgG4-Related Disease: A Case Report of Obstructive Seminal Vesiculitis

**DOI:** 10.7759/cureus.98772

**Published:** 2025-12-08

**Authors:** Hafs Elhag, Jonathan McAdam, David Curry

**Affiliations:** 1 Urology, Belfast Health and Social Care Trust, Belfast, GBR

**Keywords:** igg4 disease, obstructive uropathy, pelvic mass, seminal vesicle, urology case report

## Abstract

Immunoglobulin G4-related disease (IgG4-RD) is a systemic fibroinflammatory condition known to form "pseudotumors" that mimic malignancy. We previously reported the case of a 59-year-old male who presented with acute kidney injury from a unilateral ureteric obstruction, diagnosed as right-sided seminal vesiculitis. We now report the crucial clinical follow-up. During surveillance, the patient developed a new, contralateral pelvic mass and presacral thickening. A PET-CT scan confirmed high metabolic activity, raising suspicion for malignancy. Serological testing was definitive, revealing a markedly elevated serum IgG4 level of 7.76 g/L (normal <1.3 g/L). A CT-guided biopsy of the new lesion excluded malignancy and showed a dense lymphoplasmacytic infiltrate with an IgG4/IgG ratio >40%. The diagnosis was revised to multifocal IgG4-RD. The patient was commenced on Rituximab, which led to a marked improvement in both his serological markers and associated joint symptoms. This case demonstrates that a focal urological finding like seminal vesiculitis can be the novel debut presentation of a systemic IgG4-related disease, highlighting the need for a high index of suspicion for systemic inflammatory conditions in cases of atypical or evolving urological masses.

## Introduction

Immunoglobulin G4-related disease (IgG4-RD) is a chronic, immune-mediated fibroinflammatory condition characterised by a dense lymphoplasmacytic infiltrate and a tendency toward storiform fibrosis [1]. A key feature of IgG4-RD is its propensity to form mass-like lesions or "pseudotumors" that can mimic malignancy, leading to diagnostic challenges [2]. While IgG4-RD can affect nearly any organ, its urological manifestations are well-documented, including retroperitoneal fibrosis, ureteritis, and IgG4-related prostatitis [3].

Seminal vesiculitis is rare and usually caused by infection, often occurring alongside prostatitis. Treatment typically involves a course of appropriate antibiotic therapy, although severe cases complicated by abscess formation may require percutaneous drainage to clear the infection [4]. However, if the condition does not respond to antibiotics or presents as an unusual solid mass, clinicians must look for other causes, including malignancy or systemic inflammation.

We previously reported the case of a 59-year-old male who presented with an acute kidney injury from a unilateral ureteric obstruction, secondary to a solitary functioning kidney. The cause of the obstruction was identified as an inflammatory mass of the right seminal vesicle, and a final diagnosis of seminal vesiculitis was made at that time [5]. We now report the crucial follow-up and diagnostic evolution in this patient, whose condition was subsequently revealed to be the first manifestation of a systemic, multifocal disease.

## Case presentation

**Figure 1:**
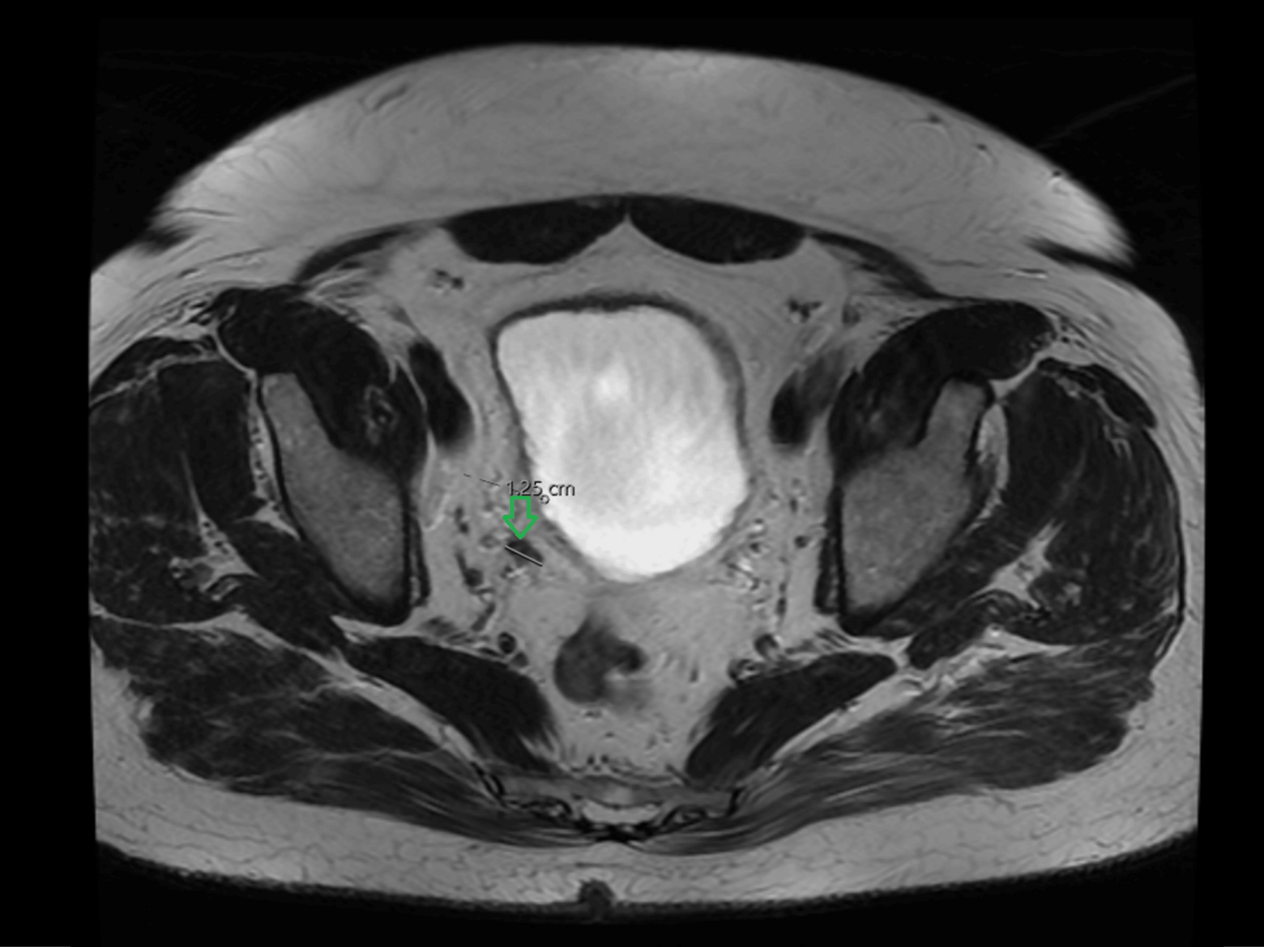
MRI Pelvis (March 2023) Axial T2-weighted image from 2023 showing the original right-sided soft tissue inflammation (green arrow), which was stable but persistent at surveillance, measuring 1.25 cm.

Following the initial event, the patient was managed with urological surveillance. An MRI in March 2023 showed right-sided soft-tissue inflammation, diagnosed as seminal vesiculitis, which was stable but persistent, measuring 1.2 cm (Figure 1).

**Figure 2:**
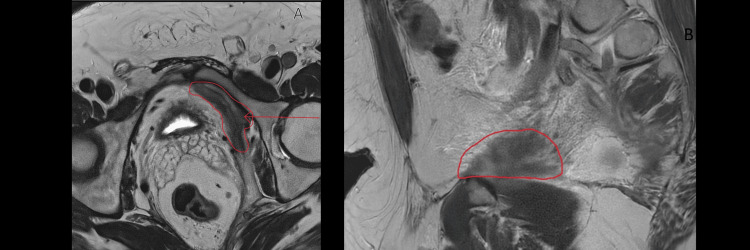
MRI Pelvis (January 2025) This scan identified new, multifocal disease. (A) Axial T2-weighted view shows the new left pelvic sidewall mass (outlined in red). (B) Sagittal T2-weighted view shows the associated abnormal thickening (outlined in red).

In January 2025, a follow-up MRI of the pelvis was performed to query resolution. This scan identified a new finding: a large, abnormal soft tissue lesion along the left pelvic sidewall, measuring 4 cm x 1.4 cm in the coronal plane and approximately 7 cm in the anteroposterior dimension. The radiologist noted it demonstrated "low signal on T1 and T2 and shows restricted diffusion", concluding that "malignancy cannot be completely excluded" and recommending multidisciplinary team discussion (Figure 2).

**Figure 3:**
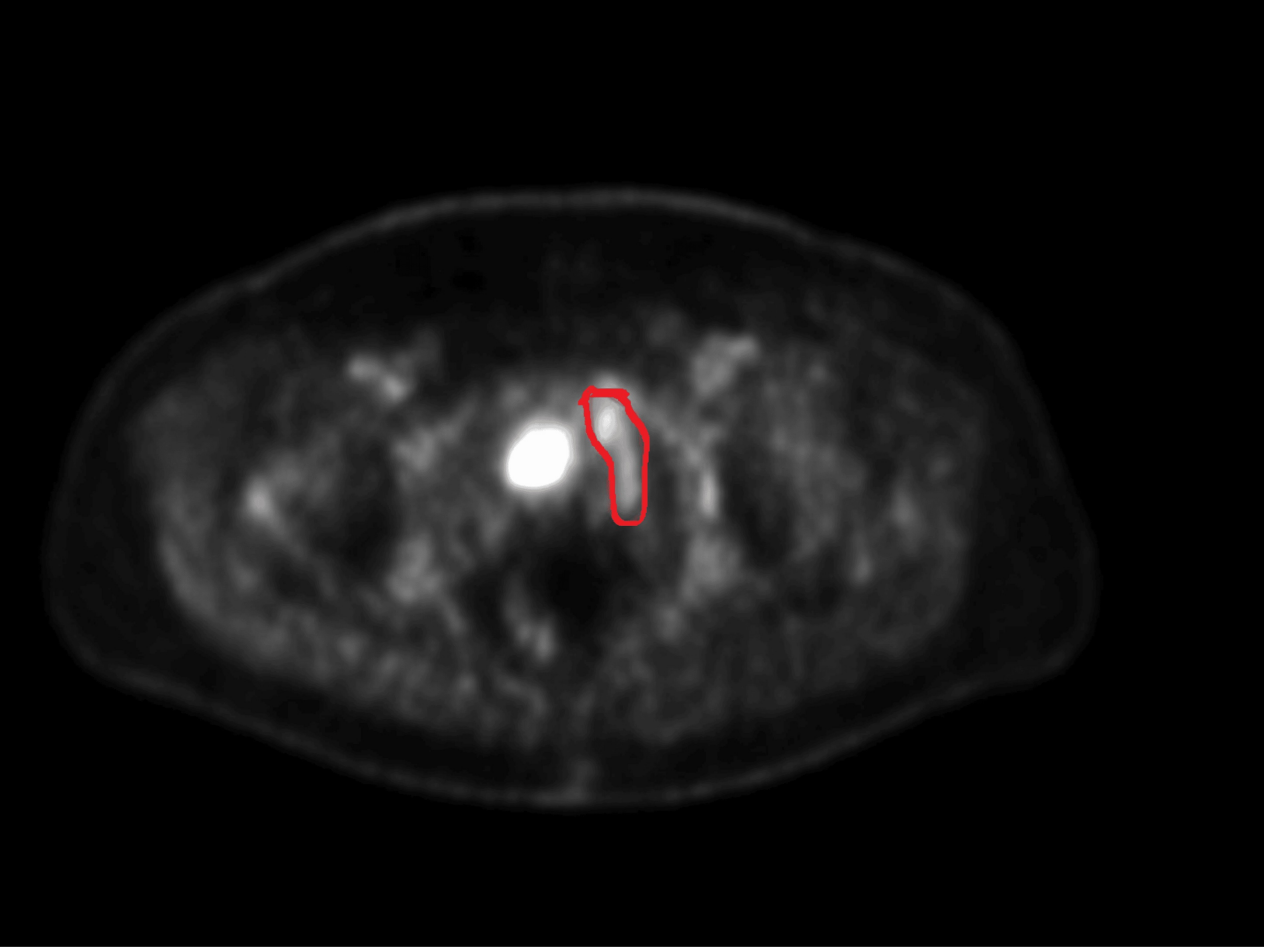
FDG PET-CT scan (February 2025) Axial PET image shows moderate, focal fluorodeoxyglucose (FDG) uptake (metabolic activity) in the left pelvic region (outlined in red), corresponding to the new mass identified on the MRI and raising suspicion for a metabolically active process.

This finding triggered a full systemic workup. A whole-body PET-CT scan was performed on February 14, 2025, using fluorodeoxyglucose (FDG) as a radioactive tracer. It revealed moderate, heterogeneous metabolic uptake in the new left pelvic sidewall lesion and in a separate area of presacral thickening (Figure 3). The differential diagnosis included lymphoma, retroperitoneal fibrosis, or IgG4-RD.

**Figure 4:**
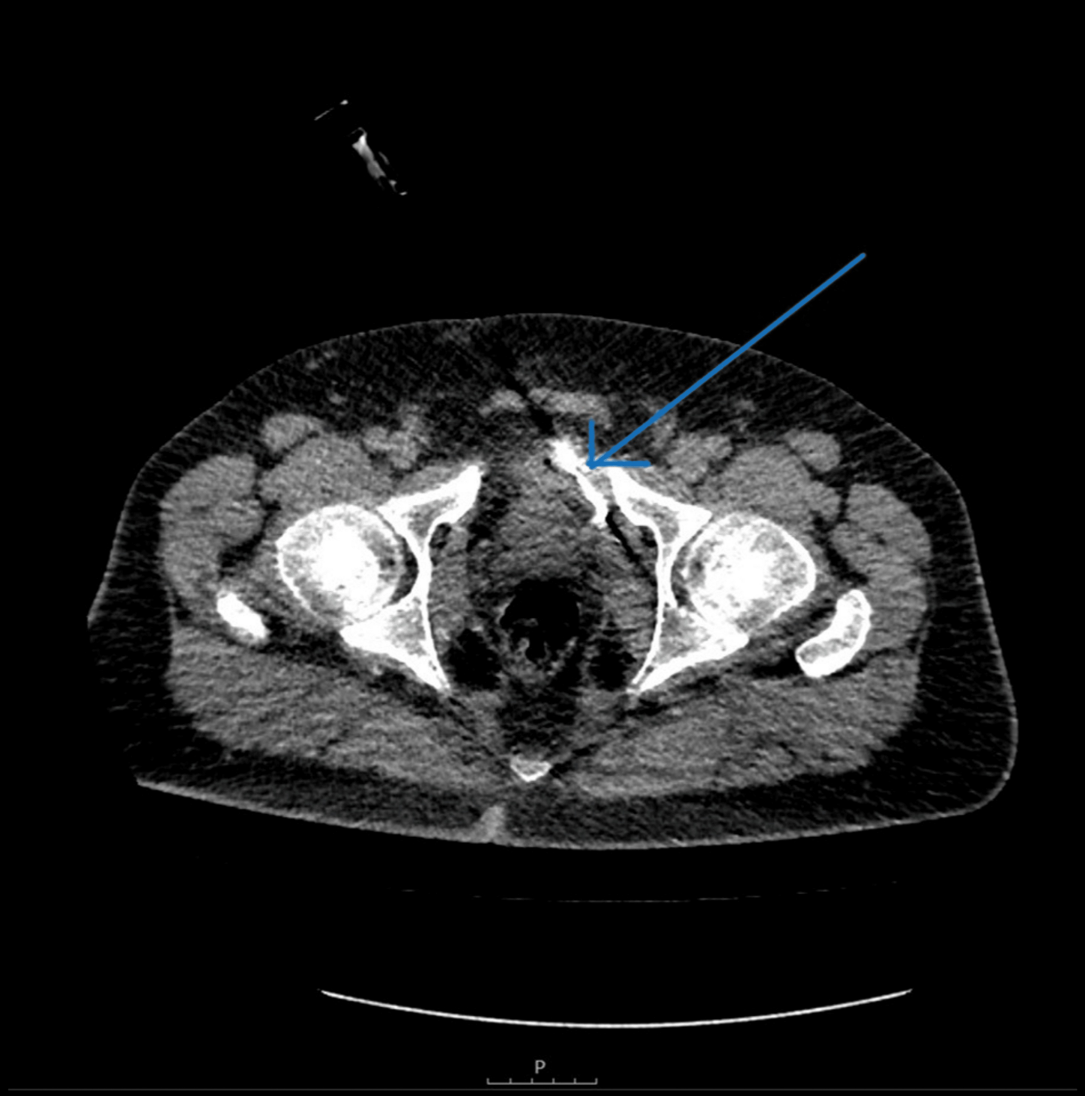
CT-guided biopsy (February 2025) Axial CT image showing the biopsy needle (blue arrow) advanced into the left-sided pelvic mass to obtain a core sample for histopathological diagnosis.

**Figure 5:**
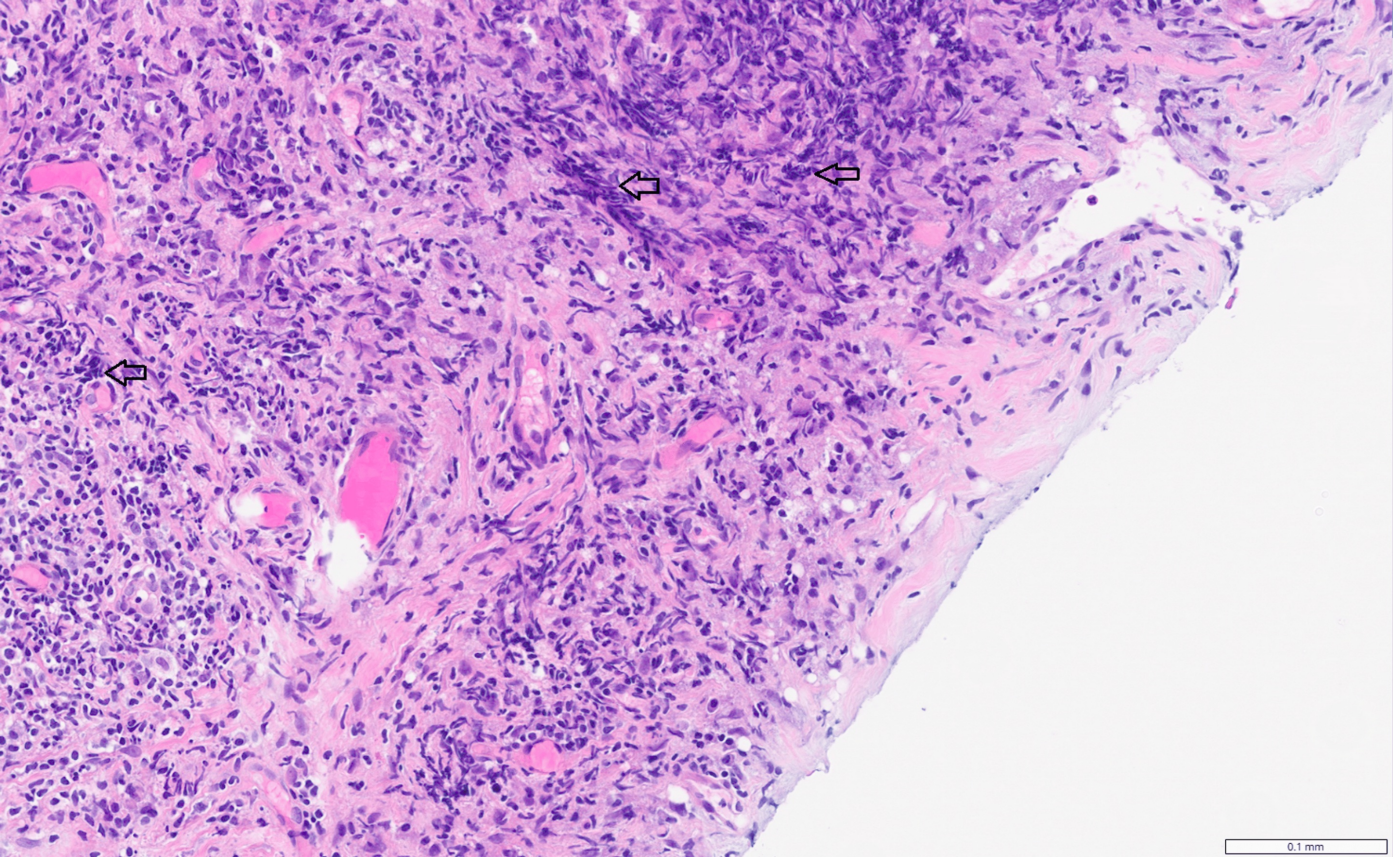
Hematoxylin & Eosin (x200 magnification) High-power view of the biopsy, showing a dense, fibroinflammatory process. The sclerotic stroma (pink) is heavily infiltrated by a lymphoplasmacytic infiltrate, which is rich in mature plasma cells and lymphocytes (regions indicated by black arrows).

**Figure 6:**
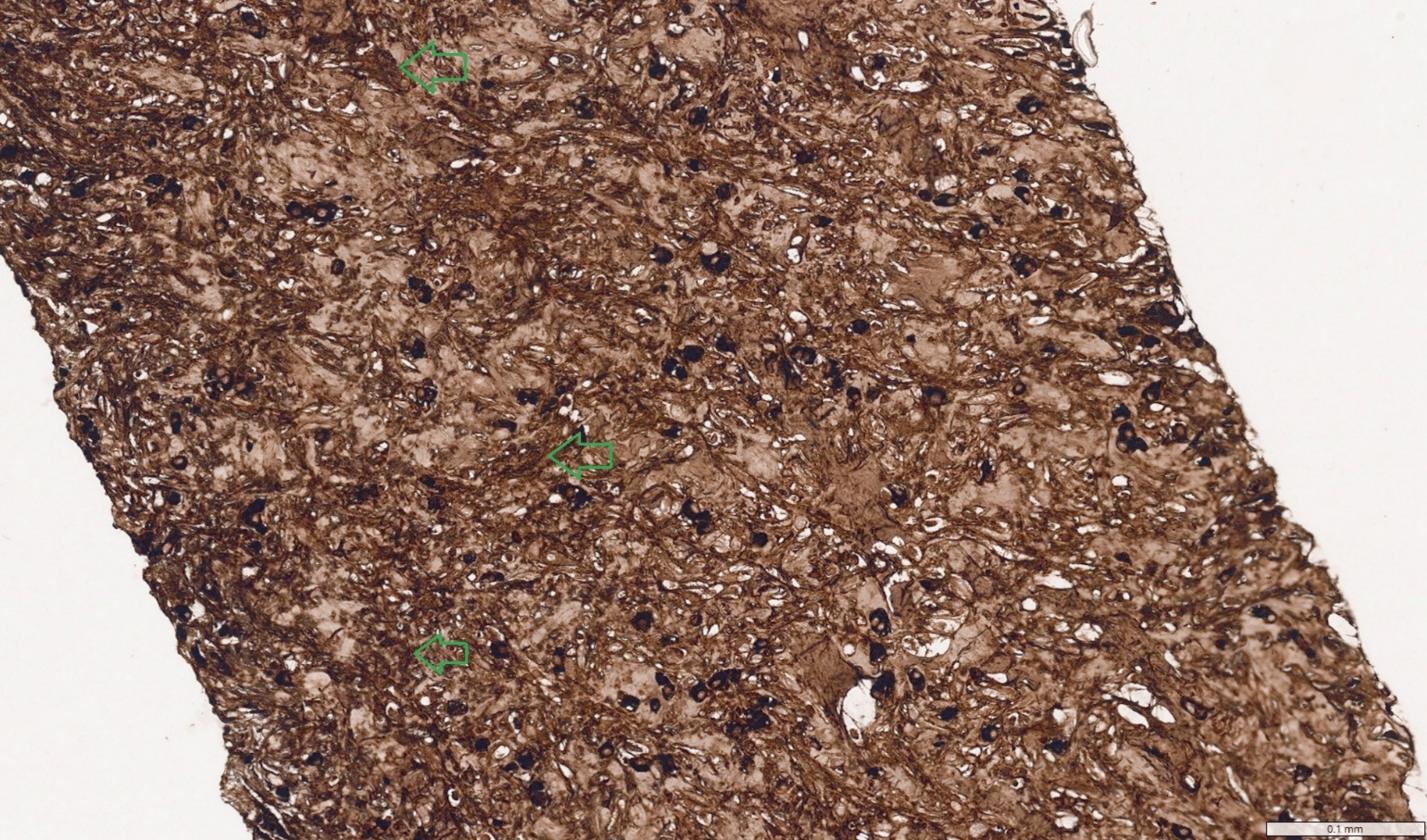
IgG (total) stain (x200 magnification) Immunohistochemistry for total IgG, highlighting the dense population of all plasma cells (stained dark brown). This slide demonstrates the sheer density of the plasma cell infiltrate (regions indicated by green arrows) and serves as the denominator for the diagnostic ratio.

**Figure 7:**
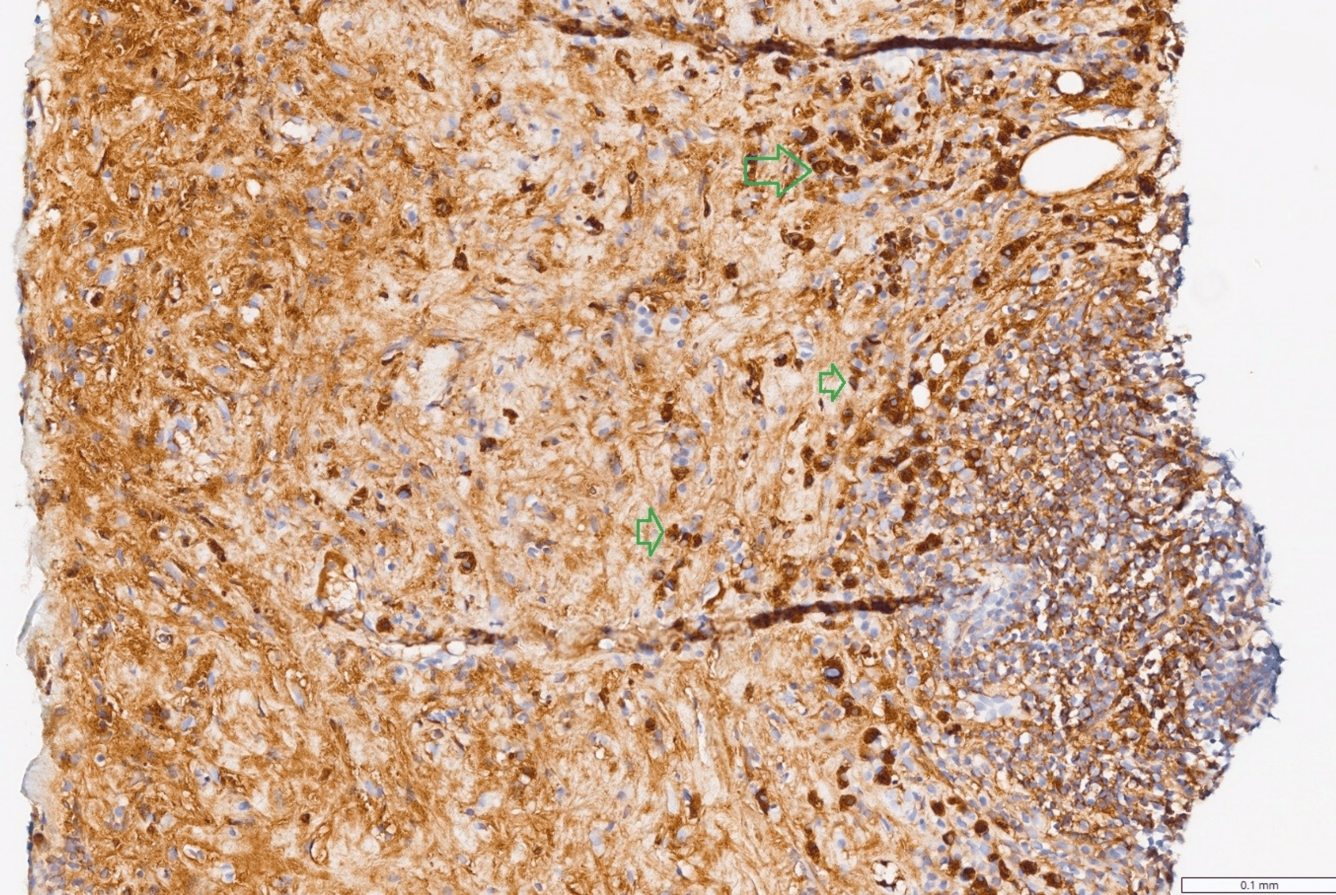
IgG4 (subtype) stain (x200 magnification) This specific stain reveals that the vast majority of plasma cells seen in Figure 6 are also positive for IgG4 (dense clusters indicated by green arrows). This visual comparison confirms an IgG4/IgG ratio >40% and was the key finding for the diagnosis of IgG4-related disease.

On February 17, 2025, the patient underwent a CT-guided needle biopsy of the left-sided pelvic mass (Figure 4). Histopathological analysis showed a dense, sclerotic fibrous connective tissue diffusely infiltrated by mature lymphocytes and plasma cells (Figure 5). An extensive immunohistochemical workup was performed to rule out a lymphoproliferative disorder (lymphoma), which was negative. Crucially, immunohistochemistry for IgG and IgG4 was performed. The slides demonstrated a massive increase in the total plasma cell population (IgG) (Figure 6), and nearly all of these cells were found to be IgG4-positive (Figure 7). The final report noted an IgG4/IgG ratio of >40%, with findings "suggestive but not diagnostic of IgG4-related disease".

Following the biopsy, the patient was referred to the rheumatology clinic. Serological testing on March 3, 2025, provided the definitive diagnosis, revealing a markedly elevated serum IgG4 level of 7.76 g/L (reference range <1.3 g/L). Additional bloodwork was significant for an elevated total IgG of 16.38 g/L (range 6-16 g/L) and a creatinine of 125 µmol/L (eGFR 53), consistent with his known chronic kidney disease. A connective tissue screen and viral serologies were negative. The key laboratory findings are summarised in Table 1.

**Table 1:** Key laboratory and serological findings before and after treatment eGFR - estimated glomerular filtration rate; CTD - connective tissue disease; IGRA - interferon gamma release assay; TB - tuberculosis

Parameter	3 March 2025 (pre-rituximab)	11 September 2025 (post-rituximab)	Reference range
Serum IgG4 (g/L)	7.76	Not repeated	< 1.3
Total IgG (g/L)	16.38	13.44	6.0 - 16.0
Creatinine (µmol/L)	125	117	59 - 104
eGFR (mL/min/1.73m²)	53	57	> 60
C-reactive protein (mg/L)*	21.6	12	< 5
CTD screen, IGRA TB, viral screen	Negative	N/A	Negative

Given the combined radiological, histological, and definitive serological evidence, a final diagnosis of multifocal IgG4-related disease was made. The patient was commenced on rituximab infusions for steroid-sparing therapy. At his eight-month follow-up review, the patient reported a significant improvement in his associated joint symptoms, and blood tests showed a reduction in his total IgG to 13.44 g/L and a slight improvement in renal function (estimated glomerular filtration rate 57), indicating a positive response to treatment (Table 1).

## Discussion

This case report presents a rare and diagnostically challenging evolution of a patient's condition, from what was initially believed to be a focal urological inflammation to a definitive diagnosis of systemic IgG4-RD. The key strength of this report is the longitudinal follow-up, which reframes the initial diagnosis of seminal vesiculitis as the novel debut of a multifocal, immune-mediated disease.

IgG4-RD is a known "great imitator", with urological involvement commonly presenting as retroperitoneal fibrosis, tubulointerstitial nephritis, or IgG4-related prostatitis, all of which can be mistaken for malignancy [6]. A literature review confirms that while some reports of IgG4-related prostatitis with urinary tract obstruction, they do not describe obstructive seminal vesiculitis as the primary, symptomatic, and focal presenting event [7,8]. To our knowledge, this is the first report to document this specific clinical presentation.

The case also highlights the real-world diagnostic pathway. The biopsy findings were "suggestive but not diagnostic" because they lacked the classic "storiform" fibrosis or "obliterative phlebitis". However, the findings of a dense lymphoplasmacytic infiltrate with an IgG4/IgG ratio >40% are, by themselves, highly specific. This aligns with the 2011 comprehensive diagnostic criteria, where such findings, combined with a high serum IgG4 and typical imaging, are sufficient for a definitive diagnosis, even without the full histological triad [2].

Finally, the patient's positive clinical and serological response to rituximab aligns with modern, steroid-sparing treatment protocols for IgG4-RD [9] and provides objective evidence that the correct diagnosis was reached and the underlying systemic disease is being appropriately managed. The management of IgG4-RD typically involves systemic immunosuppression. Glucocorticoids are the established first-line therapy for inducing remission. However, in patients with contraindications to steroids, significant comorbidities (such as chronic kidney disease and severe gout in our patient), or those at high risk of relapse, B-cell depletion therapy with rituximab is an effective alternative or steroid-sparing agent [9]. In this case, the decision to proceed directly to rituximab was driven by the patient's renal profile and personal preference to avoid steroid-associated adverse effects.

## Conclusions

We report, to our knowledge, the first case of IgG4-related disease presenting as focal, obstructive seminal vesiculitis that subsequently evolved into a contralateral pelvic mass. This case highlights that IgG4-RD can debut with highly atypical, organ-specific urological presentations.

Clinicians should maintain a high index of suspicion for systemic inflammatory conditions, such as IgG4-RD, when faced with new, multifocal, or contralateral inflammatory masses during surveillance, even if a prior diagnosis of localised inflammation has been made.

A high-yield diagnostic approach for such atypical pelvic lesions should include measuring serum IgG4 levels and utilising functional imaging (PET-CT) to map disease extent. Crucially, obtaining tissue samples for histological diagnosis is essential to differentiate this condition from malignancy and confirm the presence of IgG4-positive plasma cells.

The prognosis for IgG4-RD is generally favourable with appropriate immunosuppressive treatment, as demonstrated by the patient's serological and symptomatic improvement upon B-cell depletion therapy. However, as it is a chronic, relapsing condition, long-term surveillance is crucial to monitor for disease recurrence and preserve critical organ function, particularly in the urinary system.
